# Knowing when to talk? Plant genome editing as a site for pre-engagement institutional reflexivity

**DOI:** 10.1177/0963662521999796

**Published:** 2021-04-03

**Authors:** Robert D.J. Smith, Sarah Hartley, Patrick Middleton, Tracey Jewitt

**Affiliations:** The University of Edinburgh, UK; University of Exeter, UK; UK Research and Innovation, UK

**Keywords:** genome editing, institutional reflexivity, public engagement, research funding organisations, science policy

## Abstract

Citizen and stakeholder engagement is frequently portrayed as vital for socially accountable science policy but there is a growing understanding of how institutional dynamics shape engagement exercises in ways that prevent them from realising their full potential. Limited attention has been devoted to developing the means to expose institutional features, allow policy-makers to reflect on how they will shape engagement and respond appropriately. Here, therefore, we develop and test a methodological framework to facilitate pre-engagement institutional reflexivity with one of the United Kingdom’s eminent science organisations as it grappled with a new, high-profile and politicised technology, genome editing. We show how this approach allowed policy-makers to reflect on their institutional position and enrich decision-making at a time when they faced pressure to legitimate decisions with engagement. Further descriptions of such pre-engagement institutional reflexivity are needed to better bridge theory and practice in the social studies of science.

## 1. Introduction

Contemporary science policy is defined by its concern with the place of public and stakeholder voices in shaping the direction and form of research and innovation trajectories. This concern manifests not only through broad trends in public administration, such as commitments to ‘Open Government Initiatives’ (Fung, 2015; Pallett, 2015), but is also mirrored by innovation-specific developments that include the normalisation of calls for engagement around new and emerging technologies (Rose, 2012), institutionalisation of state experiments with engagement in science policy (Pallett, 2015), and broad uptake of governance concepts such as responsible innovation (Stilgoe et al., 2013). Soliciting public input, it is argued, increases accountability for research funding and bolsters policy-makers’ capacity to align scientific and public values (Bozeman and Sarewitz, 2011).

Engagement forms vary substantially and are contingent on a multitude of institutional dynamics (Bickerstaff et al., 2010; Fung, 2015). The literature is replete with examples of attempts to foster participation in science policy (e.g. Bellamy et al., 2016; Betten et al., 2018; Entradas, 2014; Macnaghten, 2020) but these examples must be set against studies of the ‘mess’ of policy-making that show how path dependency and lock-in are inherent to institutionalisation processes within this domain (Dunlop, 2010; Marris and Calvert, 2019). Through institutionalisation, particular rules, practices and ways of conceptualising problems become formally mandated and informally normalised (Frickel and Moore, 2006). When faced with new situations to navigate, policy-makers frequently fall back on these entrenched ways of thinking (Bickerstaff et al., 2010; Dunlop, 2010), meaning engagement is likely to be conducted using methods and thought patterns that occupy only a small area of the possible ‘institutional design space’ (Fung, 2015).

Inferred as necessary – but not explored in detail – within much of the science policy literature, is a reflexive process policy-makers must go through as they explore possible design choices for engagement exercises (Bryson et al., 2013; Fung, 2015; Stilgoe et al., 2014; Wynne, 2006). Clark (2018: 372), for instance, argues that common participation design guidelines should be augmented with an initial step, ‘one that looks inward instead of outward’ to situate those with the power to design and commission engagement activities within a broader institutional landscape. This process of ‘institutional reflexivity’, which we define as a cross-organisational process through which policy-makers identify, examine and respond to implicit assumptions and commitments, has become a prominent concept in the sociology of science (Stirling, 2016; Wynne, 1993, 2006, 2011). In theory, institutional reflexivity would augment public policy-makers’ capacity to sense when which way of enrolling which public is most appropriate to delivering public value in diverse institutional environments (Stirling, 2008). However, institutional reflexivity has far greater theoretical than empirical delineation and as such, the process of generating reflexive organisational responses to their institutional setting is rarely articulated in a way that makes them repeatable.

This article’s central argument is that institutional reflexivity can enhance the theory and practice of engagement in science policy. It unfolds in two parts. First, it uses the institutional context of science policy in the United Kingdom and its shift towards to more participative modes of governing as an empirical context to outline the concept of institutional reflexivity and articulate its potential value. It then provides a detailed analysis of pre-engagement institutional reflexivity in one of the United Kingdom’s national research funding organisations, the Biotechnology and Biological Sciences Research Council (BBSRC), as staff sought to develop a response to a politicised and high-profile emerging technology, genome editing.

The article results from an interactive social science collaboration, required to bridge theory and practice in science policy (Whatmore, 2009). The collaboration sought to advance a process to guide decision-making when there was significant pressure to formulate strategic policy and engage widely. We demonstrate how staff generated knowledge about their organisational position through a process of pre-engagement institutional reflexivity and discuss the significance of the outcome – non-engagement – for science policy. Therefore, this article goes beyond the observation that ‘institutions matter’ in shaping engagement outcomes (e.g. Bickerstaff et al., 2010; Dunlop, 2010; Rothstein, 2013) to articulate how science policy-makers can address these entrenched ways of thinking about engagement decision-making.

## 2. Theorising the need for institutional reflexivity about engagement

Studies of science policy in the sociology of science emphasise the importance of norms, logics and rationalities, which act as informal institutions shaping action (Bickerstaff et al., 2010; Rothstein, 2013). These institutions are durable systems of ideas and practices that structure and shape social life (Frickel and Moore, 2006). They act as filters, making some practices and patterns of thought possible while simultaneously foreclosing alternatives (Bickerstaff et al., 2010; Rothstein, 2013). Institutions are created and maintained by sustained social practice and it is often during controversies that such practices may be particularly strained, allowing new institutions to coalesce (Hay, 2008). In the United Kingdom, now-routine state-sponsored engagements with emerging science and technology were seeded by crises of scientific authority around bovine spongiform encephalopathy, foot and mouth disease, bovine tuberculosis and genetically modified organisms (GMOs) at the turn of the century. These controversies propagated the creation of new political actors mandated to create deliberative processes to feed into policy advice (Hajer, 2003). The United Kingdom’s first consensus conference took place in 1993, convened by BBSRC on plant biotechnology (BBSRC and The Science Museum, 1994), and 2002–2003 saw a landmark engagement exercise *GM Nation?*, in which British citizens debated the potential commercialisation of GMOs (Horlick-Jones et al., 2006).

Since these watersheds, science policy-makers in UK Research Councils have held over 16 substantial public engagement exercises (Macnaghten and Chilvers, 2014). Government bodies formalised engagement in science by establishing, for instance, definitions of Public Dialogue (Pallett and Chilvers, 2013). Through this process of institutionalisation, however, nascent forms of policy formulation were tailored to fit with entrenched ideas about the public interest, decision-making authority and hierarchies of evidence, so that engagement became ‘domesticated’ and less able to challenge entrenched norms within UK science policy-making (Rothstein, 2013). Three deeply held institutional norms have coalesced in this process of domestication. They shape engagement by prioritising certain practices over others and operate in consort to restrict new ways of thinking about why, when and with whom to engage. They are, therefore, particularly important to unpack.

The first norm is the belief that continued scientific/technological advancement is contingent on the maintenance of public acceptance and trust, which must be actively sought through engagement (Frow, 2018; Wynne, 2006). The European Academies Science Advisory Council’s (EASAC), 2017, position report on genome editing is archetypal: ‘There has to be trust between scientists and the public, and, to build trust there has to be public engagement’ (EASAC, 2017: 3). This norm enacts an instrumental rationale: engagement is primarily a method to ensure scientific and innovation trajectories are supported and can be represented as credible without deferring decision-making power to those being consulted (Stirling, 2008; Wynne, 2006). It means that engagement activities do not need to be connected to decision-making. As such, it forecloses alternative rationales (e.g. to produce knowledge) for which engagement in science administration may be pursued (Fung, 2015).

The second institutional norm is for science policy engagement to frame a discussion about what a convened ‘mini-public’ thinks about (e.g. to what degree it accepts) a single technology (e.g. synthetic biology) in a neutral setting (Bogner, 2012). Future scenarios are often constructed to encourage people to situate themselves in relation to the technology (Bellamy and Lezaun, 2017). Clear examples of this norm are BBSRC’s Bioenergy Public Dialogue (BBSRC, 2013) and the Royal Society’s Genetic Technologies Public Dialogue (HVM, 2018), which explore public support for single technologies. In the United Kingdom, this engagement format and framing has been replicated for decades, producing remarkably stable results that can be characterised as conditional support for science, with high levels of trust towards scientists but ambivalence about the motivations of private industry (Macnaghten and Chilvers, 2014). However, focusing engagement on a single technological solution to a pre-defined problem assumes the technology being developed is pre-determined, excludes alternative technologies or politically negotiated solutions (Helliwell et al., 2017) and fails to account for the ways in which people encounter technologies in their everyday lives (Betten et al., 2018). It closes down discussion of the cultures producing science and technology, but these aspects are often at the root of participants’ concerns (Macnaghten and Chilvers, 2014).

The third institutional norm is that engagement exercises are considered a method to generate a particular kind of evidence – opinion – that can be fed forward to policy-makers (Lezaun and Soneryd, 2007). This norm is attached to dominant epistemological judgements about the status, proper location and valid forms of evidence that circulate within science policy networks (Smallman, 2018). It also underpins a separation of public engagement (to gauge balanced public opinion) from stakeholder engagement (to gather opinions from representative interest groups) within science policy and forces participants to play particular roles – such as a ‘disinterested lay person’ or a ‘critical opponent’ – during engagement activities (Michael, 2009). For instance, in focus groups that formed a part of *GM Nation?*, disengaged lay public participants were selected for their neutrality and people with an active interest in GM technology were avoided (Horlick-Jones et al., 2006). Of course, individuals can be engaged to generate or contribute knowledge, not just opinions (Fung, 2015; Whatmore, 2009). But if engagement is designed to generate opinions rather than pluralistic knowledge, artificial dichotomies may be created, and policy-makers may remain ignorant about the presence of relevant knowledge beyond narrowly defined expert domains (Wynne, 2006).

### Institutional reflexivity and engagement

Rules and norms are vital to stable governance and trustable modes of working because they allow policy-makers to fall back on established methods when faced with new situations with high levels of uncertainty and ambiguity (Dunlop, 2010). This is especially important in the context of emerging technologies, which are inherently uncertain and ambiguous (Stilgoe et al., 2013). However, depending on incumbent structures means that those incumbent structures are the ones most likely to be furthered (Frickel and Moore, 2006). In science policy – both in the United Kingdom and other Organisation for Economic Cooperation and Development (OECD) nations (e.g. EASAC, 2017; Sarewitz, 2015) – a consequence of these norms is the formation of a particular loop: the development of a new technology alongside claims about its transformational capacity, followed by calls for structured public discussion and debate (Rose, 2012). Rarely considered within this loop are questions that situate the technology in a longer trajectory of social change, reflect on how the purportedly needed ‘debate’ might occur, and consider how its outcomes might be embedded within science administration (Macnaghten and Chilvers, 2014).

Institutional dynamics, therefore, present clear barriers to organisational learning, and the development of new designs and practices, over time (Rayner, 2004). Engagement exercises thus risk merely reiterating long-demonstrated findings about public perceptions of emerging technologies without providing routes to fully embed those lessons in science policy. Despite a deep understanding of the institutional dynamics shaping engagement outcomes (Baldwin, 2019; Bickerstaff et al., 2010; Wynne, 2006), it remains unclear how policy-makers might consider these dynamics in real time and modulate their actions accordingly. Wynne (1993, 2006, 2011) has long argued that such a gap could be filled if science policy-makers operationalised the concept of institutional reflexivity.

In contrast to moments of reflection that are common to professional life, institutional reflexivity is methodical, collective and targeted (Stilgoe et al., 2013). It aims to enable groups of people, often incumbent in positions of power, to examine the institutional configurations shaping their decision-making, consider the assumptions that sit behind said configurations and understand their impact on practice, and explore how alternative assumptions and commitments may produce different practices (Stirling, 2016). Through a process of institutional reflexivity, these groups consider the appropriateness of existing rules and norms, which have developed over time, to a new situation, moderating their actions accordingly (Wynne, 2006). Importantly, institutional reflexivity must avoid triviality through a direct connection to action; there must be a policy window or decision at stake (Wynne, 2011).

In the context of engagement in science policy, institutional reflexivity would require cross-organisational reflection on the assumptions, commitments and routine practices guiding decision-making about engagement *before* the activity’s form is determined. However, there are few empirical cases of institutional reflexivity in science policy and none in relation to decision-making about engagement. The remaining sections outline and critically engage with BBSRC’s operationalisation of this concept during 2015–2016 through the design of a method for pre-engagement decision-making.

## 3. Science policy, genome editing and engagement

National research funding councils are the preeminent site of science policy. They set priorities for their respective fields, provide science advice and contribute to national debate on topics relating to science and technology. We use the case of BBSRC, a national research funding council responsible for non-clinical biosciences and biotechnology funding in the United Kingdom, with a 2016/2017 budget of £417 million (BBSRC, 2017). This scientific remit stretches from agricultural sciences, through human and animal health, to industrial biotechnology (BBSRC, 2019).

Like other funding councils, BBSRC’s portfolio is delivered through a combination of unsolicited project proposals and large-scale strategic investments that align with its quinquennial strategic plans. The UK research councils outline ‘priority areas’ – roughly equating to grand challenges – in their strategic documents. When this research was undertaken, BBSRC had four priority areas, which had been in existence for over a decade: Industrial Biotechnology and Bioenergy, Agriculture and Food Security, Bioscience Underpinning Health, and Exploiting New Ways of Working (BBSRC, 2010).

BBSRC’s organisational structure roughly mirrors its strategic planning documents, and policy-makers are guided by a cluster of Strategic Advisory Panels. Major decisions are agreed by an Executive Group and Council of prominent individuals in the life sciences. At the time of this research, public and stakeholder engagement was built into BBSRC’s operational activities through the Communications and Engagement Team and the Bioscience for Society Strategic Advisory Panel, a committee of external experts providing insight, oversight and critical reflections on BBSRC policy, actions and future societal issues. The Communications and Engagement team had spent over a decade commissioning, convening and coordinating public and stakeholder engagement activities on topics in the biosciences, including ageing, animal health, bioenergy, the role of industry, stem cells and synthetic biology. Consequently, it not only understood the challenges of designing engagement in response to emerging science and technology but also possessed the internal legitimacy and tacit knowledge needed to sense when it might be possible to enrol the organisation into a new approach.

In 2014, genome editing, a cluster of new techniques to make modifications in organisms’ genetic sequences, emerged as a high-profile and policy-relevant issue. Many laud genome editing as revolutionary (Travis, 2015), but GM crops remain one of Europe’s most publicly contested technologies. Genome editing rekindled discussion about the role of genetic modification in social life and this debate proceeded down familiar fault lines. In Europe, early engagements centred on whether genome-edited organisms would fall inside the scope of Directive 2001/18/EU on the Deliberate Release of GMOs. There are potentially high financial costs for technology developers if genome-edited crops are classified as GMOs (Lusser et al., 2011), but these costs must be balanced against consumer choice, environmental impacts and their detection, and compatibility with alternative forms of agriculture (Helliwell et al., 2017). Traceability mechanisms have been central to European GMO regulation (Lezaun, 2006), but such mechanisms may be disrupted by genome editing.

This debate demonstrates how genome editing is simultaneously technical and political (Sarewitz, 2015) and highlights the challenge of BBSRC’s position in the research landscape. As the United Kingdom’s primary public funder of plant biotechnology, it has a responsibility to foster and support the bioscience community to deliver research outcomes. Equally, biotechnology’s public history means BBSRC is acutely aware that its actions can influence public trust and the generation of public value in the biosciences. These multiple responsibilities do not automatically align; instead they must be negotiated, demarcated and built with guidance from advisory panels, engagement exercises and written policy statements that clarify its responsibilities in contested situations.

Globally, calls for public debate about genome editing were quick to emerge from prominent scientists, entrepreneurs, social scientists and science policy organisations, such as the US National Academy of Sciences and the UK Nuffield Council on Bioethics (NCB, 2016). BBSRC’s initial response to genome editing and these calls for engagement followed a pattern similar to past technologies, such as nanoscience and bioenergy. It first convened a working group of external experts and held a workshop to develop an externally facing position statement, *New Techniques for Genetic Crop Improvement* (BBSRC, 2014b). Like previous position statements on synthetic biology and bioenergy, the genome editing position statement was cautiously optimistic about the value of plant genome editing and explicitly called for ‘active engagement of a wide range of stakeholders’ to identify how genome editing could be used in a way that accommodated multiple perspectives to deliver potential benefits (BBSRC, 2014b: 3). BBSRC subsequently established an internal cross-office working group (COWG) to act as a forum between teams responsible for strategic policy, engagement and the funding of its priority areas. Containing senior staff from across the research council, the COWG was BBSRC’s primary operational space for discussion on how to conduct the engagement called for in the position statement.

## 4. Research design and methodology

An existing relationship between BBSRC’s Communications and Engagement Team (Jewitt and Middleton) and scholars (Hartley and Smith) created an opportunity to co-design an interactive social science study (Hardy and Williams, 2011). Through collaboration, we aimed to strengthen the methodologies available to practitioners for pre-engagement decision-making and co-produce social scientific knowledge of institutional reflexivity in science policy. The Communications and Engagement Team recognised plant genome editing – and the formation of the COWG in particular – as an opportunity to learn from past experience with public dialogues and operationalise new social science ideas of value to the wider organisation. Therefore, we designed a theoretically informed methodology (Clarke and Star, 2008) that could guide BBSRC policy-makers through a decision-making process about how, with whom and ultimately when to pursue engage in the context of plant genome editing.

### Embedding institutional reflexivity in pre-engagement decision-making

**Figure 1.**
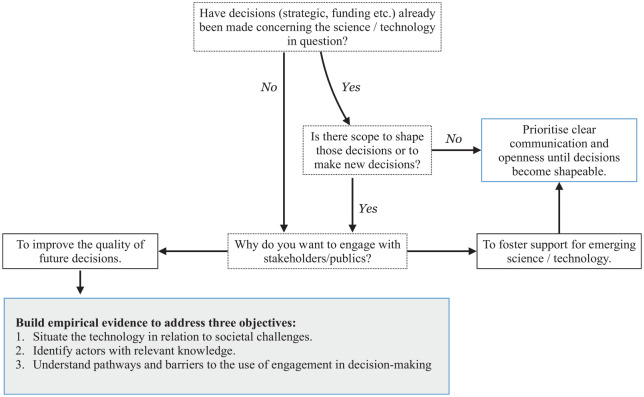
Process to operationalise institutional reflexivity within pre-engagement decision-making, embedding learning from previous engagement exercises with science and technology, as agreed upon by project team and BBSRC’s cross-office working group.

The pre-engagement process (represented in Figure 1) has two major components. The first is a short decision tree that asks policy-makers to consider the reasons for engagement. In the institutional context of UK science policy, it acts as an initial stage-gate to ensure it is possible to move beyond an instrumental rationale (i.e. engagement to build trust) by prioritising a substantive rationale for engagement, that is, engagement on the basis of knowledge generation (Stirling, 2008). This component was particularly useful for BBSRC because although the position statement on genome editing demonstrated a commitment to engagement, the form of engagement and the decisions such engagement might shape remained open and unclear. The second component is empirical and triggered if it appears possible for engagement to improve the quality of future decisions within the organisation. The empirical analysis is mixed method, with three objectives. We describe each objective and the methods employed below.

#### Objective 1: Situate the technology in relation to societal challenges

The first objective is to ascertain the focus of discussion for the engagement activity in a way that allows participants to challenge the framing adopted by policy-makers. In the context of UK science policy, this involves enabling discussion about the roles science and technology could play in addressing particular societal challenges without taking the socio-technical frame established by technologists as a foregone conclusion.

A socio-technical frame includes judgements about appropriate purposes of a technology, understandings of a social or environmental problem’s cause, as well as assumptions about how technology will interact with the environment (Betten et al., 2018; Helliwell et al., 2017). In engagement activities, socio-technical frames can be interrogated by constructing scenarios but this is too resource-intensive for a pre-engagement process (Bellamy and Lezaun, 2017; HVM, 2018). Portfolio analysis provides a faster alternative to assess the envisaged relationships between science and society (Wallace and Rafols, 2016). It is at the funding application stage that scientists must succinctly locate their research in a societal, industrial or environmental context to demonstrate its value.

To begin internal discussion about the possible trajectories of plant genome editing and the frames it might fit within, we drew on BBSRC’s funded grants portfolio (to May 2016) to analyse the claims of BBSRC-funded researchers. Data consisted of 1500-word grant summaries that include an abstract, technical summary and impact summary. BBSRC’s publicly available portfolio analyser^1^ and in-house search strings for genome editing were used to obtain text, returning 74 funded non-duplicated grants.^2^ We removed false positives (e.g. tal*ent* rather than TAL and edit*orial* rather than edit) and those not referring to plants, leaving a total of nine plant-related genome editing texts for analysis, spanning seven different organisations with the first grants starting in 2014.

Adopting a discourse analytic orientation to attend to what text *does* rather than *is* (Lamerichs and Te Molder, 2003), we subjected grant summaries to a two-phase iterative process of data reduction and data complication (Coffey and Atkinson, 1996). First, we gathered the following information from texts: competing and complementary claims about the intended action of a scientific project or technology; the framing of a societal challenge and the impacted actors. We then grouped texts to produce coherent categories in the form of societal challenges and examined the uncertainties and assumptions underpinning such claims. We supplemented this analysis with information from interviews, described below. The results were interrogated and expanded upon in a 2-hour workshop convened with the COWG.

#### Objective 2: Identify actors with relevant knowledge

The second objective is to identify participants for engagement. Responding to the norms driving engagement in science policy, actors are sought not to produce a balanced range of opinions, or on the interests they represent, but for their knowledge that is relevant but absent from the organisation’s current understanding of the way science and society might interrelate.

**Figure 2.**
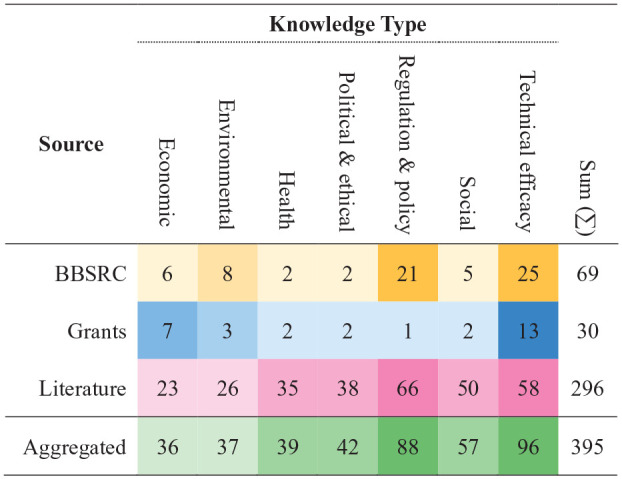
Distribution of knowledge types identified from three sources, BBSRC, grants and literature. BBSRC: Biotechnology and Biological Sciences Research Council. Each number represents an individual holding the type of knowledge (individuals can hold more than one type of knowledge). The final column shows that literature provides access to a significantly larger range of knowledge than the other two sources. The heatmap overlay indicates concentrations of knowledge for each source (e.g. BBSRC favours regulation and policy, plus technical efficacy). Aggregated sources offer the most even distribution of knowledge, showing the strength of a multi-method approach.

**Figure 3.**
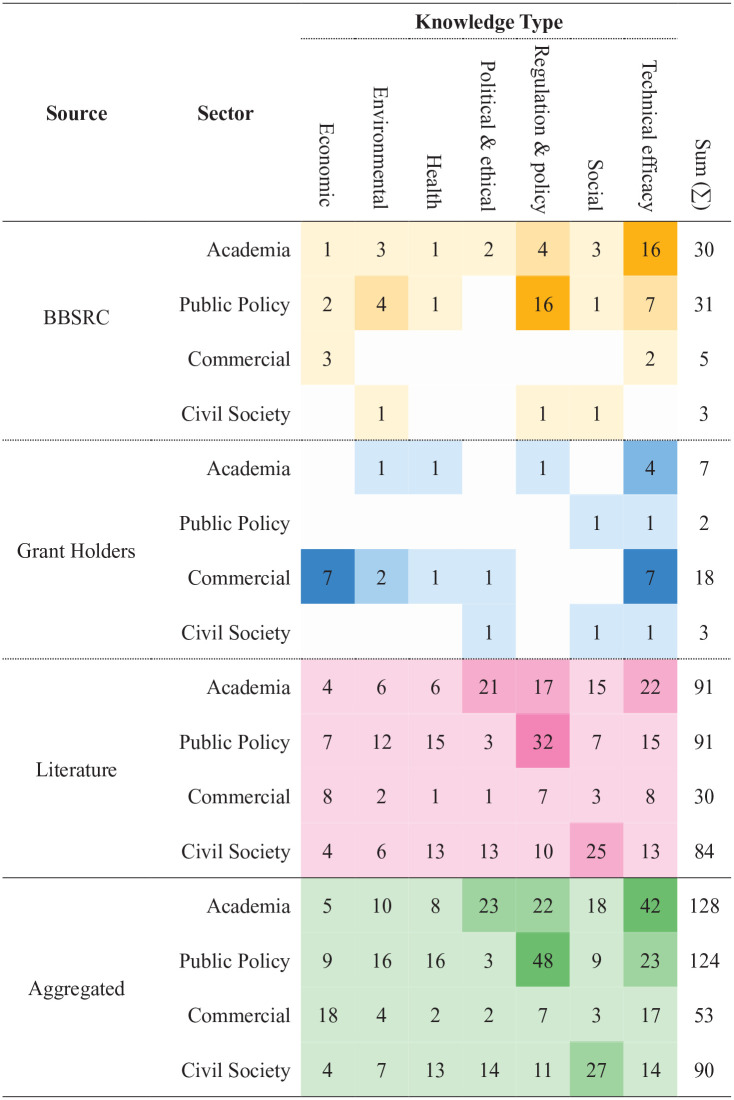
Breakdown of knowledge type by sector and source (e.g. BBSRC favours from technical knowledge from academia and regulatory/policy knowledge from public policy). BBSRC: Biotechnology and Biological Sciences Research Council. Heatmaps are specific to the source.

To identify these actors, we conducted a rapid, systematic mapping across the societal challenge areas identified in Objective 1 (Bainbridge et al., 2011; Farquharson, 2005). The types of relevant knowledge were derived from established technology appraisal processes, such as multi-criteria mapping (Bellamy et al., 2016) and bespoke tools, such as BBSRC’s *Synthetic Biology Deliberation Aid* (BBSRC et al., 2015). The knowledge types identified were economic, environmental, health, political and ethical, regulation and policy, social, and technical efficacy. Interviews and text analysis were used to produce a base map, which was supplemented with snowball sampling from grey literature and policy discussions (ACRE, 2015; BIS, 2013; HoCS&T, 2015; Lusser et al., 2011; NCB, 2015, 2016; NCB and Sciencewise ERC, 2016). Organisations, and where possible individuals, were identified from each source and the knowledge they held was then mapped against the knowledge types, producing the information in Figures 2 and 3.

#### Objective 3: Understand pathways and barriers to the use of engagement in decision-making

If engagement is to be more than a trust-building performance, it must connect to decision-making. The third objective is, therefore, to create space for policy-makers to explore how engagement could be aligned with strategic decision-making and examine the possible barriers to such an alignment.

In addition to the project team’s meetings, we conducted 13 open-ended interviews with 14 staff members and hosted discussion in two COWG meetings. Staff were mid-senior level and drawn from diverse sections of the organisation, including those responsible for portfolio analysis, strategic policy, business development, public engagement and programme management. Interviews covered four topics: organisational background; including past activities and future decisions; reasons for engagement; where outside knowledge could be useful and which internal actors might be needed for change; and technical developments in genome editing. We encouraged reflection on the existence and obduracy of past decisions within the organisation, desires and capacity for external knowledge to improve them and potential routes to achieve this. This organisational context is particularly important because it will fundamentally shape how the organisation uses the results of engagement. Key individuals within BBSRC would need to be enrolled into new engagement designs and, ultimately, see value in the results.

## 5. Preparing for engagement on plant genome editing at the BBSRC

Departing from the genome editing position statement, the Communications and Engagement Team first responded to questions in the decision tree, confirming that no strategic decision had been made about how to support genome editing; BBSRC had only supported unsolicited funding applications. However, staff were considering whether and how to develop a funding strategy for genome editing, which would be a suitable target for engagement to inform. Senior staff were open to new engagement approaches, emphasising BBSRC’s goal of placing ‘science and societal need at the centre of the discussions and to use its position statement to engage and encourage dialogue with a wide range of stakeholders’ (BBSRC, 2014a: 1). Similarly, Bioscience for Society Strategic Advisory Panel members recommended engagement should be broad, conducted alongside technology development and connect to decision-making (BBSRC, 2015b). On the basis of these early discussions, BBSRC executive staff mandated the Communications and Engagement Team to develop an approach to engagement, ‘defined as two-way and with the explicit aim of informing BBSRC’s thoughts, actions or policies and strategies’ (BBSRC, 2015a). Thus, the stage-gate was passed; the empirical study began.

### Situating the technology in relation to societal challenges

We found that BBSRC-funded scientists locate plant genome editing in relation to five challenge areas: industrial biotechnology and bioenergy, biology underpinning health, genetic tool development, food security and sustainable agriculture. In total, 10 specific problems (e.g. novel antibiotics; disease resistant plants) were identified within these challenges. Of the five challenge areas, food security and sustainable agriculture were the most prevalent with seven grants focused exclusively on these two challenges.

Here, all grant summaries claimed the societal challenges of food security and/or sustainable agriculture could be addressed through a resulting technical product – a plant with new traits. Behind these scientific claims are particular visions of agriculture, and assumptions about the relationship between genome editing and agricultural practices (Doubleday and Wynne, 2011). For instance, implicit within the pest management grants were assumptions that host-pest relationships would remain controllable following disruption of a host-pest pathway, disrupting pathways would reduce the use of agrochemicals; and removing pests is necessary for the propagation of sustainable agriculture. Researchers thus prioritised one vision of food and agricultural systems and how genome editing might address them.

The potential scientific trajectories were reported in detail but pathways to addressing societal challenges were largely broad and unspecific, suggesting many of the visions invoked in association with the technology were aspirational rather than concrete and assumed to flow from technological advances. This is a common and well-documented feature of scientific discourse, understood by sociologists as a key aspect of scientific practice; non-specific promises and broad visions provide orientation points for scientific communities to coalesce without being held accountable for them in the future (Stone, 2017). These claims emerge from social situations and are often based on scientists’ assumptions about what is valued by the funder. There is, therefore, a corresponding amount of social agency on the part of the funding organisation to shape the norms leading to the production of such claims, perhaps more so than is commonly assumed (Schot and Steinmueller, 2018).

### Identifying actors with relevant knowledge

The mapping process revealed that BBSRC’s previous engagement activities had favoured scientific and policy knowledge from academia and public policy, while BBSRC-funded researchers favoured economic and technical knowledge from industry actors (Figures 2 and 3). Grey literature produced a more even distribution, with slight skews towards regulatory and governance, technical/feasibility and social. Aggregating sources (Figure 3, final category) demonstrated the value of a multi-method approach. While combined sources leant heavily towards academics with technical knowledge, at least one actor was provided for each knowledge type and a plurality existed in all but two.

We also saw variations in specificity (individuals vs organisations) as well as significant biases towards commercial (funded researchers) and public policy (BBSRC staff) sectors vis-à-vis actors identified using grey literature. For instance, grant proposals collectively identified a wide range of actors, spanning users (e.g. industrial partners, farmers, food retailers, horticulturists, foresters and seed breeders), field managers, regulators and policy-makers, and risk assessors. However, these users were generally non-specific categories rather than individuals or organisations. Science museums and publics were included, but generally as conduits and recipients of scientific knowledge, respectively, rather than holders of knowledge that could feed into engagement activities.

This process thus complemented BBSRC’s nascent understanding of plant genome editing trajectories by expanding the gaze beyond dominant expert domains. Our multi-method approach, combining three data sources, identified more actors (158 vs 44) with a wider breadth of knowledge than was previously the case. It provided access to actors across four sectors (academia, public policy, commercial and civil society) who hold the knowledge needed to interrogate key assumptions about the relationships between science, society and the environment within BBSRC’s research portfolio.

### Understanding pathways and barriers to the use of engagement in decision-making

Interviews both reaffirmed BBSRC’s desire to be responsible, its commitment to engagement, and identified three routes between an engagement activity and decision-making, each with respective challenges. One route would be to target the work and membership of BBSRC’s strategic advisory panels. Some senior staff viewed panel membership as an attractive option because they typically sought individuals with broad, interactional expertise commensurate with the breadth of knowledge identified in the actor mapping process. However, this approach was double-edged: BBSRC would begin to enact long-term structural changes to its advisory processes but such a reconfiguration would be a significant undertaking, requiring the support of the Executive Board and Council. It was unclear whether a concern with genome editing engagement could drive such a change. The simplest and most attractive route between engagement and decision-making was to directly connect an engagement activity to BBSRC’s strategic priority setting processes. These operated at two scales, the largest being the development of an overarching strategic vision involving input from across the organisation, its strategic panels and occurring on a loose quinquennial cycle. Operating at a smaller scale were sector-specific priority areas, overseen by specific departments and developed in consort with external experts.

Several aspects of priority setting would complicate the path of knowledge from engagement to decision-making. No organisation-wide strategic agenda setting process was planned in the near future, leaving only the smaller-scale targeted support. But here, genome editing’s status as a high-profile cluster of scientific techniques for genetic modification meant it would be unlikely to meet implicitly held criteria for such support. Staff emphasised that targeted interventions were designed to support under-resourced or diffuse areas of science that BBSRC believed had clear societal benefit. Examples of such interventions included bioenergy in 2006, synthetic biology in 2007 and industrial biotechnology in 2011. In line with established narratives of research policy (Schot and Steinmueller, 2018), staff did not want to be seen as ‘picking winners’ by adopting a technology-centric approach to priority setting. Instead, it was for scientists and peer-review panels to determine the specific ways in which BBSRC’s ‘technology agnostic’ priorities were met.

The final possible pathway was through by modifying BBSRC’s genome editing position statement. This was attractive because it was publicly framed as a ‘live document’ to be periodically revised and there was internal acknowledgement that it lacked the broad input BBSRC had committed to. However, given the challenges above, it was clear that if it were to have any impact on BBSRC’s decision-making, a bespoke route would have to be negotiated and then constructed as the engagement activity was designed. This would be a substantial undertaking without a clear outcome. We discuss the significance of these findings in the next section, which considers the consequences of BBSRC’s pre-engagement process for decision-making about engagement around plant genome editing, locates institutional reflexivity within it and situates the lessons within a broader landscape of sociology of science scholarship and practice to consider how capacity for institutional reflexivity can be built in the future.

## 6. Locating institutional reflexivity in science policy

At BBSRC, findings of the pre-engagement process were shared internally through the COWG and Bioscience for Society Strategic Advisory Panel. They were accompanied by the following recommendations. First, any engagement around genome editing should centre on the mutual trajectories of science, technology and society, placing at its heart BBSRC’s desire to be a ‘responsible investor on behalf of the public’. Second, BBSRC should seek to engage broadly, using the knowledge types (Figures 2 and 3) identified in the pre-engagement process to identify knowledge gaps and challenge assumptions in the organisation. Third, staff should explore how the two extant pathways with direct links to decision-making, advisory panel activities and priority setting, could be opened up.

This final recommendation was made with the knowledge that connecting an engagement activity to strategic decision-making would be a significant challenge, but that without such a connection engagement should not proceed. Given the context of UK science policy and the status of genome editing in BBSRC, it was neither clear what new knowledge would be produced nor how it could instigate a substantive organisational response. Therefore, these recommendations effectively amounted to an overarching recommendation that BBSRC pause to consider how extant pathways to decision-making could be legitimately altered to incorporate a wider diversity of knowledge than they might otherwise. To date, BBSRC has not held an engagement activity on genome editing. Where, when and how, then, was institutional reflexivity fostered within BBSRC’s pre-engagement process? In demarcating the concept, we delineated three criteria that could structure a process of institutional reflexivity. First, it would be a collective process with the capacity to transect a science organisation. Second, the target of reflection would be the institutional configurations – rules, norms, assumptions and commitments – that shape policy-makers’ actions. And third, it would avoid triviality through direct connection to decision-making about a salient science policy issue. We unpack criteria one and two concomitantly before turning to the third.

Two phases of reflexivity are visible within BBSRC’s pre-engagement process: in its design and enactment. These phases involved overlapping, but distinct, collectives and foci for reflection (see Table 1). In the design phase, the collective was constituted narrowly by academics and practitioners with experience of engagement in science policy. Here, reflection was broad, internalising several critical analyses about the rationales, framing and use of engagement activities that developed over an extended timeframe. The pre-engagement process thus codified an organisational response to the norms of science policy engagement: it moved beyond a rationale based on public trust and towards one based on knowledge generation; it shifted the focus away from a single technology with assumed benefits and towards a conversation about the way technologies might interlace with societal challenges; and it sought to include people based not on their neutrality or ability to represent certain interests, but on a pluralistic understanding of expertise.

**Table 1.** Collectives and aspects of institutional reflexivity considered as part of BBSRC’s pre-engagement process.

Phase	Collective	Norms and assumptions confronted
Design	Academic authors, members of Communications and Engagement team	Broad temporal/methodological reflections: 1. Dominant instrumental rationale of prior engagement activities, performed through separation of engagement from strategic decision-making. 2. Consistent overemphasis on technological possibilities and underemphasis on social context. 3. Individuals targeted based on the ability to represent interest group or produce unbiased opinion.
Enactment	Above, plus COWG	Situational/procedural reflections: 1. Rationale for engagement (through consensus regarding method). 2. Assumptions associated with technological development. 3. Actors engaged with and to be engaged with. 4. Potential outcomes of engagement activity (whether results could be used substantively). 5. Framing of the policy problem (genome editing demands engagement) challenged.

COWG: cross-office working group.

To enact the pre-engagement process, the collective was broader, incorporating representatives of teams from across the organisation. Here, social scientific methodologies produced evidence that allowed staff to reflect on the specifics of the case, interrogating assumptions and commitments about the nature of genome editing, the actors who would be valuable to engage with, and the ways in which engagement might be used.

Interrogating the small amount of BBSRC-funded research around plant genome editing drew attention to a common framing of the relationship between bioscience and agriculture, which held that sustainable production would be achieved by developing new plant breeds. There are a range of alternative possible framings of sustainable agriculture (Helliwell et al., 2017; Vanloqueren and Baret, 2009), and it is only through exposition that this assumption becomes amenable to debate. However, even within this shared frame, the diversity in this relatively small portfolio exposed policy-makers to choices about the trajectories of plant genome editing; within a shared goal of crop improvement, there was variation about whether genome editing would produce transgenic plants or whether it would be used to develop markers for traits that can be produced in existing breeding programmes. These are different trajectories for plant genome editing with distinctive regulatory and socio-political consequences, showing that even within the shared agricultural frame, BBSRC’s support of the technology was not a simple binary choice; it would be legitimate for a strategic funding policy to grapple with such issues, and then respond to them through funding call design, for instance, by prioritising particular innovation pathways or definitions of sustainability (Wynne, 2011). While this capacity has long been theorised within the sociology of science (Schot and Steinmueller, 2018), it is rarely demonstrated to science policy-makers in ways that are tangible, highlighting a strength of policy experiments designed in collaboration between practitioners and scholars.

Through actor mapping, the group confronted biases in the kinds of knowledge BBSRC and its funded scientists deemed relevant to plant genome editing; to date, participating actors were dominated by technical plant science from academia (i.e. BBSRC’s scientific community). While public sector actors with political and legal expertise were included, a much broader range of relevant political-economic experts had been excluded. And while social and environmental knowledge was present, it was under-represented in relation to the aforementioned technical emphasis. Juxtaposing these maps with those derived from relevant literature significantly broadened range of knowledge available to BBSRC, offering alternatives to these commitments for future engagement activities.

In advanced liberal democracies, such as the United Kingdom, twenty-first-century science has been animated by a concern that it must be conducted within the bounds of public trust; if trust is lost, controversy may ensue and scientific research will be curtailed (Frow, 2018). Engagement exercises are used to show science aligns with mainstream public opinion, demonstrating trustworthiness, thus providing autonomy. When we began this collaboration, the policy issue driving BBSRC’s actions was ‘given a desire to engage broadly around plant genome editing, how should the organisation proceed?’ Such a question fits squarely within the broader framing of engagement. The need for engagement is assumed and the institutional dynamics that will shape it are not adequately considered.

BBSRC’s pre-engagement process coupled reflection and decision-making about engagement to reflection and decision-making about genome editing funding policy. By following this process, considering the nature of the technology and its social context in parallel, staff identified that genome editing would *not* be an appropriate candidate for strategic funding policy. Therefore engagement, were it to proceed, would likely only enact an instrumental rationale. Instead, the pre-engagement process identified that the most appropriate sites for engagement would be extant priority setting processes and the work of advisory panels. Staff, therefore, began to reframe this policy problem, de-emphasising the question of what an engagement exercise for plant genome editing should look like and refocusing on how priority setting and decision-making could draw on broader arrays of knowledge than they currently do.

Answering these questions would require a process of organisational transformation. However, such a transformation is only likely in the medium-term future because it lies outside the policy problem that gave the initial impetus to act. As such, this policy window produced a methodology for institutional reflexivity about engagement but another policy window will be needed to make the changes to BBSRC’s organisational configuration that staff deemed necessary to reimagine the place of engagement within science policy.

In this instance, institutional reflexivity effectively foreclosed the possibility of public or stakeholder engagement occurring, furthering the scientisation of policy (Weingart, 1999). This is an uncomfortable outcome for advocates of collaborative governance and deliberative democracy. However, the quantity of engagement must be balanced with building policy-makers’ capacity for better quality engagement, that is, moving away from an institutional landscape that produces instrumental forms of engagement and towards a landscape that produces normative or substantive forms of engagement with real opportunities to foster civic capacity (Fung, 2015; Stirling, 2008). This means understanding when the right moment to engage is and when it might not be, knowing when to talk and when not to (Stilgoe et al., 2014).

This situation emphasises two related needs. First is the need for continued and expanded networks for scholar–practitioner interactions. One notable feature of this study is the established relationships between practitioners and academics, developed in a broader organisational context of BBSRC making space to consider the societal dimensions of the science it supports, and academics analysing the workings of these processes. Within this context, we developed tacit knowledge of the institutional setting, the challenges staff in BBSRC faced, and what it might take for the organisation to give meaning to a concept like institutional reflexivity. It seems clear that such relationships would be necessary to capitalise on similar moments in the future. Second, there is a need for more studies detailing comparable acts of translation in a range of contexts. While engagement is endemic to contemporary science policy, there are relatively few detailed accounts of how concepts, such as institutional reflexivity – and the methodologies enacting them – travel (c.f. Soneryd, 2016). It, therefore, remains unclear what concepts might be allowed to mean in different institutional settings, and how the learning generated in one translational exercise might be built on in other settings.

In the past decade, scholars have called for a research agenda attentive to the institutions and processes that shape the practice of science policy, and the ways in which they produce or circumscribe the creation of public value (Joly, 2015; Stirling, 2016; Wynne, 2006). Such institutional dynamics may pose democratic challenges, in that legitimate voices are effectively excluded from participation (Baldwin, 2019; Clark, 2018; Dawson, 2018), or they may hinder learning, in that an engagement exercise is likely to simply duplicate findings from prior activities without considering how they can influence practice (Macnaghten and Chilvers, 2014; Smallman, 2018). By employing institutional reflexivity as a design criterion for a pre-engagement decision-making process in science policy, this article goes further, demonstrating how theories from the sociology of science can contribute to the practice of policy-making. Through methodical processes designed to foster institutional reflexivity, norms can be rendered open to scrutiny and new administrative practices can be inscribed.

## Notes

1.
https://bbsrc.ukri.org/research/grants-search/

2. Search strings: gene OR genome AND edit*| crispr OR ‘genome editing’ OR ‘gene editing’ OR ‘gene edits’ OR ‘genome edits’| ‘zinc fingers’ OR ‘ZFN’ OR ‘TALEN’ OR ‘TALENS’ OR ‘TAL endonuclease’. No date restrictions were set, and both completed and current grants were returned. For example, duplicates occur (1) when the same project is submitted as two proposals for geographically distinct and (2) when people move institutions.
